# Efficient Solution for Large-Scale IoT Applications with Proactive Edge-Cloud Publish/Subscribe Brokers Clustering

**DOI:** 10.3390/s21248232

**Published:** 2021-12-09

**Authors:** Van-Nam Pham, Ga-Won Lee, VanDung Nguyen, Eui-Nam Huh

**Affiliations:** Department of Computer Science and Engineering, Kyung Hee University, Yongin-si 17104, Korea; nampv@khu.ac.kr (V.-N.P.); gawon@khu.ac.kr (G.-W.L.); ngvandung85@khu.ac.kr (V.N.)

**Keywords:** topic-based publish/subscribe, implicit collaborative filtering, topic similarity, topic subscription prediction, distributed publish/subscribe systems, Internet of Things, broker overlays

## Abstract

Large-scale IoT applications with dozens of thousands of geo-distributed IoT devices creating enormous volumes of data pose a big challenge for designing communication systems that provide data delivery with low latency and high scalability. In this paper, we investigate a hierarchical Edge-Cloud publish/subscribe brokers model using an efficient two-tier routing scheme to alleviate these issues when transmitting event notifications in wide-scale IoT systems. In this model, IoT devices take advantage of proximate edge brokers strategically deployed in edge networks for data delivery services in order to reduce latency. To deliver data more efficiently, we propose a proactive mechanism that applies collaborative filtering techniques to efficiently cluster edge brokers with geographic proximity that publish and/or subscribe to similar topics. This allows brokers in the same cluster to exchange data directly with each other to further reduce data delivery latency. In addition, we devise a coordinative scheme to help brokers discover and bridge similar topic channels in the whole system, informing other brokers for data delivery in an efficient manner. Extensive simulation results prove that our model can adeptly support event notifications in terms of low latency, small amounts of relay traffic, and high scalability for large-scale, delay-sensitive IoT applications. Specifically, in comparison with other similar Edge-Cloud approaches, our proposal achieves the best in terms of relay traffic among brokers, about 7.77% on average. In addition, our model’s average delivery latency is approximately 66% of PubSubCoord-alike’s one.

## 1. Introduction

Along with 5G network advantages, Internet of Things (IoT) technologies connect different sensors and devices on a large scale so communications, sharing, and vital actions can take place when needed [1]. There are various applications in many domains, such as agriculture, healthcare, transportation, industrial automation, and smart homes [2]. However, the IoT poses many challenges because future systems will be more complex, with huge amounts of data and ever-increasing demands from applications. On the other hand, sensors and devices create unprecedented amounts of data that need to be transferred, stored, and analyzed in the proper places. One known solution is for data primarily generated near the edge of the network to be processed by applications deployed in edge networks to provide fast reaction times. Consequently, several supporting technologies, such as cloud computing, fog computing, cloudlets, and information-centric networks, have been utilized and deployed to bring computing and storage capabilities closer to the end user [3,4].

Importantly, IoT applications require high usability and low processing system resource usage. For example, sensor data often need to be integrated with other data in order to observe spatio-temporal dependencies to infer more complete knowledge for the user [5]. In city environments, traffic data from sensors need to be processed in real time. Consequently, the abundance of sensors and their frequent sampling rates create continuous data that can be voluminous [6]. In addition, in heterogeneous signed social networks, people share many pieces of content on a daily basis. It also needs a method to detect the user’s topics of interest, such as ComPath [7] based on their interests/disinterests to measure the similarities. In this paper, we consider large-scale IoT applications as the IoT systems involving thousands of IoT devices and spreading over large geographical areas such as cities inside a country [8,9]. Therefore, the challenge is designing a dynamic data delivery solution for large-scale IoT applications that can provide data dissemination with low latency and high scalability.

The publish/subscribe (pub/sub) paradigm (PSP) is one known solution for dynamic large-scale applications because it provides a scalable and suitable interaction scheme [10]. In this communication paradigm, publishers (producers) advertise and publish events via publications; subscribers (consumers) express their interest in events via subscription, and receive relevant notifications generated by publishers. Note that both publications and subscriptions are sent to an event manager. Upon arrival, the event manager identifies all matching subscriptions, notifying interested subscribers. In addition, participants in PSP are decoupled in space (not knowing each other) and in time (not active at the same time) and are not synchronized (publishers are not blocked during event production; subscribers can be asynchronously notified) [10].

On the other hand, the PSP has proven to be a powerful communications protocol for developing a wide range of distributed applications. In addition, this paradigm can support useful and flexible features, such as anonymity, many-to-many communications, and asynchronicity for distributed systems [10]. With these helpful characteristics, the PSP has been used in many complex, large-scale information diffusion systems, such as Spotify, Twitter, and Facebook  [11]. Take Spotify [12] as an example; it utilizes the PSP to provide various features for social interactions. Users can subscribe to topics under the Friends, Playlists, and Artists pages. Whenever users subscribe to an artist’s page, they receive notifications about that artist, such as new album releases. Unsurprisingly, the PSP and especially topic-based pub/sub (TBPS) systems have been deployed mainly for data dissemination in many extensive IoT systems [13,14,15]. Specifically, in an essential setup, a lightweight messaging protocol such as MQTT [16] supports connectivity between IoT devices and applications. In addition, a message broker/server is employed to facilitate data exchange among IoT devices. Through this facility, IoT applications access the collected data and control the devices via the server by using APIs. However, this method is only suited to LAN environments and does not scale up when applied to wide-scale IoT projects where a very large number of geo-distributed IoT devices and a large number of brokers exist [17]. Another approach is centralized in the deployment of pub/sub brokers in the cloud; however, this strategy will increase the propagation delay in distributed IoT applications. Therefore, the geo-distributed deployment of Edge-Cloud pub/sub brokers is a promising solution that can take advantage of low latency and extensive computing or storage resources. Nevertheless, we need to cluster these edge brokers properly to save network bandwidth and to reduce transmission latency. Consequently, there is an evident desideratum to develop advanced coordination schemes to organize and scale these brokers effectively.

To cope with these issues, we designed a hierarchical Edge-Cloud pub/sub brokers model to support data delivery in large-scale IoT projects, offering low latency and high scalability. In addition, we introduce a two-tier routing scheme so the brokers can efficiently notify subscribers about events. The main contributions of this paper are summarized as follows:

- We apply implicit collaborative filtering techniques to predict future topics for pub/sub brokers in order to proactively cluster them into proper groupings.
- We design a two-tier routing scheme for the clusters, which includes intra-cluster and inter-cluster procedures to enhance end-to-end data delivery to IoT devices.
- We devise a coordinative scheme so pub/sub brokers can collaborate in linking all topic channels in the system efficiently.
- Extensive simulation results indicate that our proposed model can support data dissemination for large-scale IoT applications, offering low latency, small amounts of relay traffic, and high scalability.

The remainder of the paper is structured as follows. Section 2 presents related works on pub/sub systems that can support large-scale IoT projects. Section 3 outlines the system model of our proposal; we also explain the topic recommendation procedure for pub/sub brokers, and we describe the process model of hierarchical pub/sub brokers for data delivery. In Section 4, we present in detail the coordination procedures to bridge joint topics among broker clusters. We provide simulation results to validate our claims in Section 5, and in Section 6, we conclude the paper.

## 2. Related Works

Publish/subscribe schemes can be classified into three main variants: topic-based, content-based, and type-based [10]. Of these schemes, the topic-based pub/sub scheme, which classifies events into topics, is suited for real-time IoT service scenarios because it does not need too much run time overhead [18].

A merging of IoT and artificial intelligence was introduced in [19]—a three-level model with different requirements: a smart city and IoT infrastructure, fog computing, and cloud computing. Rathore et al. in [20] proposed an edge-based aggregation-and-processing strategy for raw data by forwarding them through gateways to smart city applications using the Internet. The authors in [21] discussed IoT data edge-processed aggregation, network communication, and service deployments in terms of the potential benefits and impacts. In addition, the authors in [22] presented a smart routing solution for crowdsourcing data with mobile edge computing (MEC) in smart cities using reinforcement learning (RL) for intelligent network communication resources. Moreover, in [23], the authors presented the PSIoTRL framework, which includes an intelligent orchestrator for IoT management to address the issue of transferring an enormous volume of IoT data through a network with constrained communication resources.

Cross-platform messaging solutions, such as Firebase Cloud Messaging (FCM) [24] and IoTivity [25], can play the role of development platforms for IoT applications. FCM allows application developers to send notifications or data messages to users through IoT applications that need to collect data and deliver low-latency content. For sending and receiving messages, an implementation of FCM comprises two main components: a trusted server environment that supports the FCM server protocols to build, target, and send messages and client applications that receive messages. IoTivity is another famous framework to support the ever-increasing demands of IoT systems. The ultimate goal of this open source project is to create a new standard for connecting billions of wired and wireless devices. In this framework, data are collected from IoT devices and finally stored in the IoTivity Global Cloud using pub/sub brokers. However, this mechanism might cause high latency in delay-sensitive IoT applications.

On the other hand, many works that design protocols and algorithms to build scalable peer-to-peer TBPS systems support message delivery in wide-scale applications [11,26,27]. One hybrid TBPS overlay system [11] allows low-relay messages among peers, and scales up well with a large number of peers and topics. Another TBPS system presented in [26] exploits topic subscription correlations among peers while dynamically clustering them on a skewed distributed hash table. Furthermore, a distributed protocol, SpiderCast [27], dynamically organizes correlated workload peers into an overlay network to support TBPS communication. However, it may be infeasible to apply such methods on IoT devices in wide-scale IoT applications due to the nature of the limited resources in IoT devices.

Building scalable broker-based TBPS systems to handle message delivery in wide-scale IoT systems is another promising approach. Dynamoth [28] is a scalable middleware platform in which independent pub/sub servers are deployed in the cloud to handle data delivery for producers and consumers in latency-constrained environments. It provides a scalable, load-balancing, topic-routing service among brokers inside the cloud for their remote clients. Due to long delays when transferring data from clients to cloud brokers, however, it cannot guarantee meeting the needs of delay-sensitive IoT applications. On the other hand, edge pub/sub brokers are placed in isolated edge networks in PubSubCoord [29]. Therefore, messages exchanged among clients belonging to the same edge network are routed through a common edge broker to reduce delays. In addition, routing servers are deployed in the cloud to route messages among edge brokers. However, in this system, how to exploit topic correlations among proximate edge brokers to further decrease delays is not clearly addressed. Furthermore, with the development of edge computing, the design of mechanisms for edge pub/sub brokers to dynamically communicate and collaborate with each other to improve system performance is an open issue. Hence, in this paper, we apply machine learning algorithms (collaborative filtering and density-based clustering algorithms) to build a broker overlay network and to design an efficient coordination scheme for distributed pub/sub brokers to transmit event notifications in wide-scale IoT systems.

## 3. The System Model

**Figure 1:**
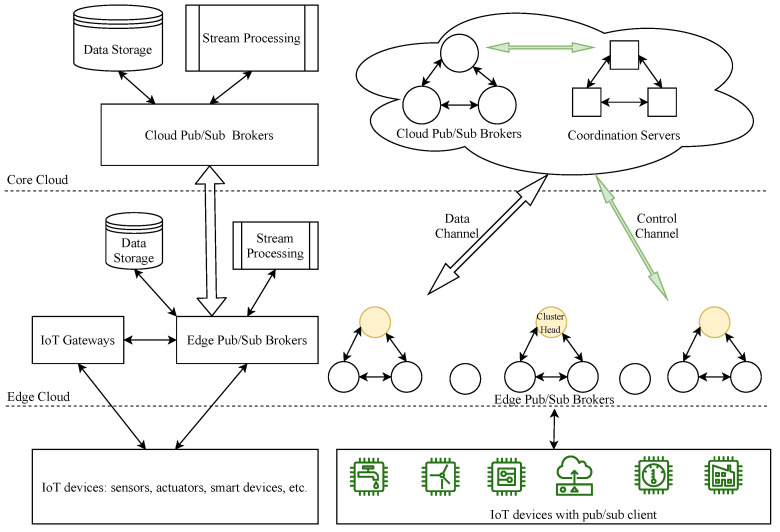
The system model.

In this paper, we consider a pub/sub brokers model used in conjunction with an essential edge-computing-based IoT architecture to enhance the data delivery capabilities for large-scale IoT systems, as shown in Figure 1. IoT devices such as sensors, actuators, and smart devices are positioned at the base layer. They periodically collect data, send them to IoT gateways or pub/sub brokers, and act accordingly. With message delivery or pub/sub client modules installed, the IoT devices are generally called IoT clients in our system; these clients can be interconnected to exchange data among themselves or provide value-added services to end users, usually via IoT gateways or pub/sub brokers with APIs provided. The IoT gateways will gather and aggregate data from IoT devices, preprocess them, and then send appropriate instructions so IoT devices can react accordingly. In addition, in a general cloud-based IoT architecture, the gateways send complicated processing tasks and forward the summarized/filtered data via pub/sub systems to cloud servers for further analytics and long-term storage [30]; they also send the processed results back to the IoT devices after receiving them from the cloud servers. By mainly relying on storage, message, and process services from the cloud, however, this architecture may incur some problems, such as high latency, excessive bandwidth consumption, and some privacy and security concerns, etc. [30]. Therefore, the integration of the IoT and edge computing can alleviate these issues by offering computation, message, and storage services in edge clouds [31]. In our model, pub/sub brokers are strategically deployed at the edge cloud tier, called edge pub/sub brokers, to aggregate data from the IoT gateways for local data storage and real-time data processing services. These brokers can also directly provide a message delivery service for IoT clients in many IoT systems, as described in [15]. Furthermore, we expand pub/sub brokers at the cloud tier, called cloud pub/sub brokers, to provide a data relay service among edge broker clusters and far-end edge brokers, or to congregate pre-processed/filtered data from edge brokers for long-term storage and deep analytics in the cloud to extract further meaningful insights. In each cluster, the cluster head is the only broker forwarding and receiving common topic data to/from a relay-cloud pub/sub broker for other interested edge brokers. This method will help to save network bandwidth consumption when transferring data among brokers and will reduce an edge broker’s node degree (the number of connections to other edge brokers needed to exchange data). In our proposed topic-based pub/sub system, each pub/sub broker maintains a control channel with a coordinator to exchange topic channel information. In short, the coordination component will keep track of information and topics that edge brokers publish or subscribe to and will coordinate all brokers to link all topic channels in the system for data delivery.

### 3.1. The Topic Recommendation Procedure for Edge Pub/Sub Brokers

In this subsection, we describe procedures to apply machine learning techniques that proactively predict with high probability future topics that edge brokers, on behalf of their clients, may subscribe to. These recommended topics, then, will be included with the brokers’ current topics to efficiently cluster the edge brokers into groups with similar topic subscriptions and proximity coordinates.

Collaborative filtering (CF) methods have been widely applied in recommender systems (RSs) [32]. CF methods provide personalized recommendations to users based on new user–item associations extracted from relationships between users and from interdependencies among items, such as products, movies, etc. [33]. There are two primary CF approaches: neighborhood models and latent factor models.

User-based neighborhood models deduce recommended items for users from a set of frequently purchased/liked items of similarly like-minded individuals. This is based on the assumption that users purchase/like items that are purchased/liked by other like-minded users. In contrast, item-based neighborhood models focus on item similarities. From there, the system suggests items that are similar to items in which the user has a high level of interest [32].

Alternatively, latent factor models, such as those produced by Singular Value Decomposition (SVD) of the user–item interactions matrix, factorize the matrix into two or more smaller matrices that represent the most useful information in a lower-dimensional space. The idea behind this approach is that there exist latent features that describe the relationships among items and users; by transforming both users and items to the same latent space, the method can reveal latent factors that explain observed user–item interactions [34]. Latent factor models offer improved prediction accuracy, flexibility, and scalability for RSs [35]. In addition, to provide CF recommendations on implicit feedback data, such as clickstreams, purchase histories, etc., that are abundant, easy to collect in many systems, and that indirectly reflect user opinion about products/items, there exist one-class CF algorithms [33,36]. With these advantages, we therefore apply collaborative filtering methods to predict future topics that clients may subscribe to at each edge pub/sub broker. Then, we add these recommended topics to edge brokers’ topic lists and perform broker clustering based on topic similarities and geolocations.

Assume that, in the system, we have a list of *M* pub/sub brokers and a list of *N* topics. The subscription matrix is denoted by $S\in R^{M\times N}$, where element $s_{bt}$ denotes the number of clients of broker *b* that subscribe to topic *t*, so $s_{bt}$ is zero if no client of broker *b* subscribes to topic *t*. As with typical SVD models [37,38], we associate each broker *b* with a broker-factors vector, $x_{b}\in R^{K}$, and we associate each topic *t* with a topic-factors vector, $y_{t}\in R^{K}$. The goal here is to factorize matrix **S** into two matrices, $B^{M\times K}$ and $L^{N\times K}$, that yield the best approximation of $S:S\approx B\cdot L^{T}$, and the approximate value of $s_{bt}$ is calculated by an inner product, i.e., $\hat{s_{bt}}=x_{b}^{T}y_{t}$. When applying SVD methods for explicit feedback datasets, we directly model only the registered subscriptions, and the model would be:

(1) $$\min_{x^{*},y^{*}}\sum_{s_{bt}\_is\_known}{\left(s_{bt}-x_{b}^{T}y_{t}\right)}^{2}+\lambda \left({∥x_{b}∥}^{2}+{∥y_{t}∥}^{2}\right).$$

Here, λ is employed to regularize the model and avoid overfitting; parameters are usually learned by gradient descent. However, in our system, it is impractical to collect explicit feedback from clients about topic preferences. Instead, we just register which brokers subscribe to which topics (i.e., represent for their clients), and we try to deal with this implicit feedback to infer future topics that brokers may subscribe to with similar likelihood. Furthermore, implicit feedback datasets have special characteristics (there is no negative feedback, they are inherently noisy, etc.) that restrain us from directly applying explicit feedback algorithms [33]. Therefore, we adapted the implicit feedback CF techniques presented in [33] to solve our recommended topic problem by introducing proper variables and modifying the problem formulation. Specifically, we bring in variable $p_{bt}$ to indicate the likelihood of broker *b* subscribing to topic *t*. The $p_{bt}$ values are calculated from the $s_{bt}$ values with confidence level $c_{bt}$:

(2) $$\begin{array}{c} p_{bt}=\left\{\begin{array}{c} 1,s_{bt}>1\\ 0,s_{bt}=0 \end{array}\right.\\ c_{bt}=1+\alpha p_{bt} \end{array}.$$

Then, we transfer optimization problem (1), and the goal is to find factor vectors $x_{b}$ and $y_{t}$ by minimizing the following loss function:

(3) $$\min_{x^{*},y^{*}}\sum_{b,t}c_{bt}{\left(p_{bt}-x_{b}^{T}y_{t}\right)}^{2}+\lambda \left(\sum_{b}{∥x_{b}∥}^{2}+\sum_{t}{∥y_{t}∥}^{2}\right).$$

With the Alternate Least Squares (ALS) approach, we fix the broker factors to optimize the topic factors, and vice versa, to solve optimization problem (3). When fixing the topic factor vectors and taking a derivative of (3), we obtain the following equation for minimizing the loss function of our brokers:

(4) $$x_{b}={\left(Y^{T}C^{b}Y+\lambda I\right)}^{-1}Y^{T}C^{b}p\left(b\right).$$

Here, Y is an N×K matrix that gathers all topic factors where values are randomly initialized and are alternately updated. $C^{b}$ is defined as the diagonal N×N matrix for broker *b*, where $C_{tt}^{b}=c_{bt}$, and vector $p\left(b\right)\in R^{N}$ stores all the preferences by broker *b*. To make the matrix multiplications much less intensive, we use the fact that $Y^{T}C^{b}Y=Y^{T}Y+Y^{T}\left(C^{b}-I\right)Y$; therefore, we have the final broker factors vector as this equation:

(5) $$x_{b}={\left(Y^{T}Y+Y^{T}\left(C^{b}-I\right)Y+\lambda I\right)}^{-1}Y^{T}C^{b}p\left(b\right).$$

Alternately, by fixing the broker factor vectors, taking a derivative of (3), and using the same technique used with the broker factors, we obtain the following equation for minimizing the loss function of our topics:

(6) $$y_{t}={\left(X^{T}X+X^{T}\left(C^{t}-I\right)X+\lambda I\right)}^{-1}X^{T}C^{t}p\left(t\right).$$

In Formula (6), X is an M×K matrix that gathers all broker factors where values are also randomly initialized and are alternately updated. $C^{t}$ is defined as the diagonal M×M matrix for topic *t*, where $C_{bb}^{t}=c_{bt}$, and vector $p\left(t\right)\in R^{M}$ stores all the preferences for topic *t*. In our experiments, we set α=15 and λ=0.1, these are common parameter settings in CF algorithms according to [32]. By repeating the process of alternately computing Equations (5) and (6) until we reach the expected matrix approximation of S, we obtain one broker factor matrix and one topic factor matrix, called B and L, respectively. These matrices give us topic-prediction scores for each broker *b* and the topic list:

(7) $$topic\_prediction\_scores=B_{b}\cdot L^{T}.$$

With *topic-prediction scores* provided for edge brokers, we recommend to each broker a list of topics with the highest predicted scores (the number of recommended topics is, say, 10% of the broker’s topic number). Then, we perform edge broker clustering based on the updated topic lists, including recommended ones, as well as their coordinates, as described in the next subsection.

### 3.2. The Hierarchical Pub/Sub Brokers Process for Data Delivery

In this subsection, we describe the data delivery process of the hierarchical pub/sub brokers, and we reveal how Edge-Cloud pub/sub brokers are coordinated to fulfill the task of transferring end-to-end event notifications for distributed IoT clients.

**Figure 2:**
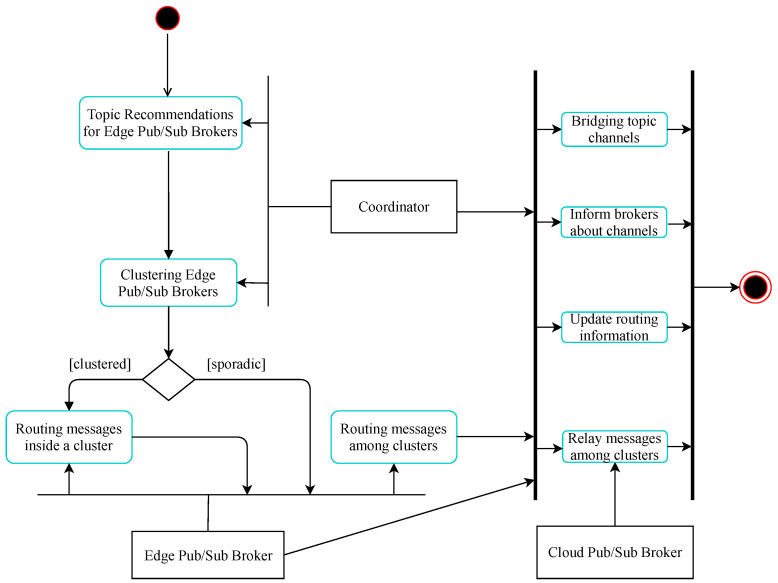
The process for hierarchical data delivery.

Assume that at time *T* the system needs to perform edge pub/sub broker clustering for data delivery enhancement. As shown in Figure 2, the coordination module of the system makes topic recommendations to edge brokers, as described in Section 3.1, and clusters these brokers with the HDBSCAN algorithm [39] into different groups based on their topic similarities. HDBSCAN is a density-based clustering algorithm that provides a clustering hierarchy from which a simplified tree of outstanding clusters can be constructed [39]. We apply this algorithm to cluster our edge brokers using cosine similarity for the pairwise distance metric between brokers, with the minimum cluster size set to five. As a result, we receive a collection of significant broker clusters and some noise, called sporadic brokers, that does not belong to any cluster. Then, these clusters are refined in another clustering round to gather only edge brokers with location proximity into a common cluster based on their coordinates. After all that, we obtain a list of clusters where edge brokers with similar topic subscriptions and that are located close to each other are put into the same cluster. In each cluster, we choose as the cluster head an edge broker where the coordinates are nearest the center of the cluster. These cluster heads will connect with their relay cloud pub/sub brokers for data delivery among clusters in the system. Likewise, sporadic brokers will exchange data on topics of common interest to other sporadic brokers or broker clusters via their relay cloud brokers.

After clustering edge brokers, coordination servers provide the edge brokers with cluster information, such as the cluster head locator (IP address and port number), cluster members’ locator, lists of joint topics, etc. This information will help edge brokers to set up connections among themselves in each cluster for direct data exchange on topics of common interest. In order to link topics that have publishers and subscribers belonging to different clusters, we select the most suitable cloud pub/sub broker for each cluster head to relay messages for that cluster, called the relay cloud broker. The best-fit cloud broker selection for each cluster head is based on subscribe utility scores between cluster head $h_{i}$ (heading up cluster *i*), and each cloud broker, $c_{j}$, in the group of cloud pub/sub brokers, *C*, as shown in Formula (8), which is inherited from a similar formula in previous work [40,41]:

(8) $$subcribe\_Utility\left(h_{i},c_{j},C\right)=\frac{\left|subs\left(h_{i}\right)\cap subs\left(c_{j}\right)\right|}{\left|subs(h_{i})\right|}-\beta *\frac{\left|subs(c_{j})\right|}{\left|subs(C)\right|}.$$

Here, $subs(h_{i})$ denotes the topic subscription list of cluster *i*, for which $h_{i}$ is the cluster head, $subs(c_{j})$ denotes the topic subscription list of cloud broker $c_{j}$, and subs(C) denotes all the subscribed topics of the cloud pub/sub brokers. In addition, $\frac{\left|subs\left(h_{i}\right)\cap subs\left(c_{j}\right)\right|}{\left|subs(h_{i})\right|}$ represents the topic subscription similarity between cluster *i* and cloud broker $c_{j}$. The more similar the subscribed topics they share, the better the topic routing efficiency in the system. The expression $\frac{\left|subs(c_{j})\right|}{\left|subs(C)\right|}$ is added to consider the workload of cloud broker $c_{j}$ in correlation to other cloud brokers. This helps to balance the load among cloud pub/sub brokers while relaying event notifications among clusters in the whole system.

With the cluster information provided, edge brokers in each cluster directly deliver data on intra-cluster topics, and they forward event notifications about inter-cluster topics to their cluster heads. In turn, the cluster heads forward the events to the assigned cloud pub/sub brokers for relay to clusters that subscribe to the topics of interest. To do that, the relay cloud broker needs to consult the coordination server for routing information. Then, the coordinator will find joint topic channels, inform the involved relay cloud brokers about the common channels bridging the topics, and will update routing information in the database.

## 4. Coordination Procedures to Bridge Joint Topics among Broker Clusters

With the establishment of the hierarchical structure of Edge-Cloud pub/sub brokers described in Section 3, our pub/sub system can efficiently provide data delivery services to geographically distributed IoT clients. Data delivery among IoT clients is performed as follows. (1) Data are organized as a collection of topics in the system. (2) In each topic channel, IoT publishers will periodically collect/sense data and send them to the responsible pub/sub brokers. (3) IoT subscribers or applications will subscribe to topic channels of interest to receive relevant event notifications via their responsible brokers. (4) Each Edge-Cloud pub/sub broker has a coordination module to communicate with a responsible coordinator in the cloud to exchange control information. (5) In turn, the coordinators will help link all topic channels from distributed pub/sub brokers in the whole system. In order to fulfill coordination tasks, the coordinators need to conduct primary interactions with pub/sub brokers as follows:

- As a new topic is published or subscribed to by IoT clients, the responsible pub/sub broker instantly informs the coordinator about the event.
- The coordination module performs the functions of clustering edge brokers, sending information about clusters to the related brokers. This information is also stored in a coordination database shared among coordination servers and is updated immediately when there are changes in the system.
- The coordination servers maintain cluster information, such as broker members, cluster heads, joint topic channels, which clusters publish and/or subscribe to which topics, and information about the clusters’ relay cloud brokers. In addition, they also maintain routing information to and from relay cloud brokers to trigger inter-cluster topic bridging when necessary.
- If a pub/sub broker receives a Publish message on a new topic, it promptly notifies the responsible coordinator to bridge with other publish brokers, if available. If publishers already exist for the topic in the same cluster as the new publishing broker, the coordinator will inform all publish brokers in the cluster about the new broker to advise them to subscribe to each other for future event notifications. If the publishing topic is new to the cluster the broker belongs to, and the topic’s publishers exist in other clusters, the coordinator will instruct all relevant relay cloud brokers to link the topic in the related clusters.
- If a pub/sub broker receives a Subscribe message on a new topic, it needs to receive all relevant event notifications on the topic from the whole system. Therefore, the broker notifies its coordinator responsible for subscription information. If the topic’s publishers are in the same cluster as the new subscribing broker, the coordinator will provide all intra-cluster publish brokers’ locators to the new broker to subscribe to the topic channel. If the subscribe topic is new to the cluster that the broker belongs to, and that topic’s publishers exist in other clusters, the coordinator will instruct the cluster’s relay cloud broker to subscribe to all related publishing clusters’ relay cloud brokers in order to receive and forward all topic event notifications.

### 4.1. Bridging Joint Publish Topic Channels among Broker Clusters

**Figure 3:**
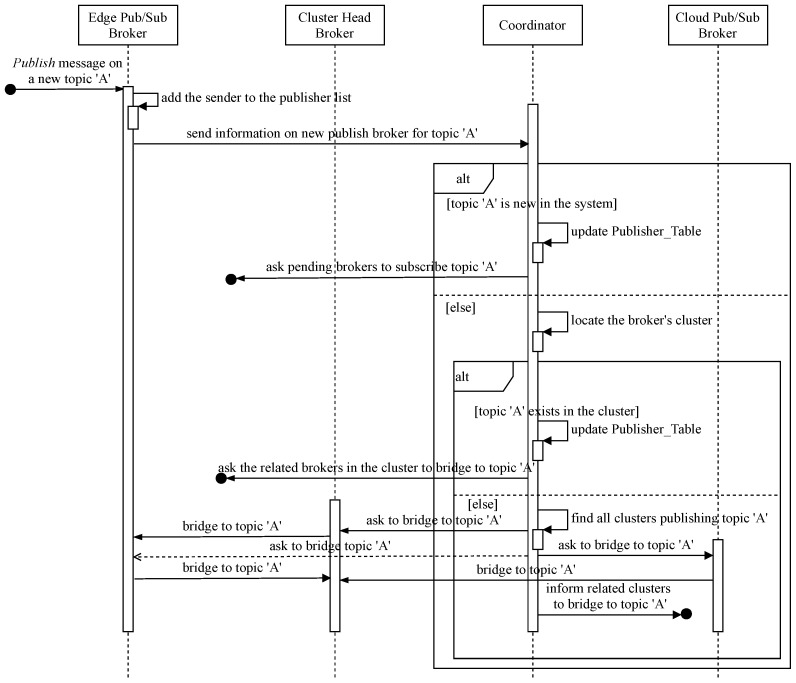
Sequence diagram for bridging joint Publish topic channels.

In our hierarchical Edge-Cloud pub/sub brokers system in Figure 1, all distributed IoT clients playing data producer or publisher for each topic channel need to be linked as a single domain to deliver event notifications on the topic in the system. Therefore, new topic publication information is provided and processed at the corresponding pub/sub broker, the cluster head, and the relay cloud broker under instructions from the coordinator. Figure 3 shows the sequence diagram for coordinative processing of a new Publish topic message when the responsible broker receives notice from its client. In addition, the coordinator implements Algorithm 1 to handle the bridging process. Table 1 lists the main variables used in Algorithms 1 and 2 and their brief descriptions; the core of the coordinative procedure is as follows:

- When a pub/sub broker receives a Publish message on a new topic, *A*, from its client, the broker adds the sender to its Publisher list and promptly informs the coordinator to bridge the topic channel if necessary.
- If topic *A* is new to the system (i.e., being the first broker to publish the topic), the responsible coordinator adds the new publish broker to the Publisher_Table and sends instructions to interested brokers in the Pending_Table, if available, to subscribe to the topic.
- If topic *A* already has publishers in the system, the coordinator needs to find out the cluster the new publish broker belongs to in order to determine whether the topic is new in that cluster or not and to retrieve the cluster’s information, such as the cluster head locator, the relay cloud broker identification, etc. If publishers of the topic already exist in the cluster, the coordinator asks the previous publish brokers to subscribe to the topic channel of the new broker, and vice versa. The coordinator adds the new member to the Publisher_Table in the coordination database. Otherwise, the informing broker’s cluster has the first publisher of topic *A*, but some other clusters may already have publishers of that topic. The coordinator needs these relevant clusters to join the new topic *A* channel. In order to do that, the coordinator looks for all clusters publishing topic *A* and navigates to their relay cloud brokers. After that, the coordinator instructs the new publish cluster to bridge to existing publish channels of topic *A*. The cluster head subscribes to topic *A* from the new broker to forward related messages to the relay cloud broker. The involved relay brokers, in turn, subscribe to topic *A* to receive and forward the topic’s event notifications.

**Algorithm 1** Bridging joint Publish event notifications among broker clusters.**Function:** coordinateJointPublishTopic(tp_Id,brk_Id,pls_Tb,pd_Tb,cls_Tb):
  1:**if** (! tp_Id**is in**pls_Tb ) **then**  2:    addPublisher(tp_Id, brk_Id, pls_Tb);  3:    **if** (tp_Id**is in**pd_Tb ) **then**  4:         pd_Scr = findPendingSubscribers(tp_Id, pd_Tb);  5:         inform_to_subscribe(tp_Id, pd_Scr);  6:    **end if**  7:**else**  8:    cls_Id, clsH_Id, rlCB_Id = findClusterInfo(tp_Id, brk_Id, pls_Tb, cls_Tb);  9:    **if** ( tp_Id**is in**cls_Id’s pls_Tb ) **then**10:         addPublisher(tp_Id, brk_Id, pls_Tb);11:         p_Brk = findIntraBrokers(tp_Id, cls_Id, pls_Tb);12:         inform_Bridging(tp_Id, p_Brk, p_Brk);13:    **else**14:         addPublisher(tp_Id, brk_Id, clsH_Id, pls_Tb);15:         rl_Brk = findInterBrokers(tp_Id, pls_Tb, cls_Tb);16:         inform_Bridging(tp_Id, brk_Id, clsH_Id);17:         inform_Bridging(tp_Id, clsH_Id, rlCB_Id);18:         inform_Bridging(tp_Id, rlCB_Id, rl_Brk);19:    **end if**20:**end if**


**Algorithm 2** Bridging joint Subscribe topic channels among broker clusters.**Function:** coordinateJointSubscribeTopic(tp_Id, brk_Id, scr_Tb, pls_Tb, pd_Tb, cls_Tb):
  1:**if** (! tp_Id**is in**pls_Tb ) **then**  2:    addSubscriber(tp_Id, brk_Id, pd_Tb);  3:**else**  4:    cls_Id, clsH_Id, rlCB_Id = findClusterInfo(tp_Id, brk_Id, scr_Tb, cls_Tb);  5:    **if** ( tp_Id**is in**cls_Id’s scr_Tb ) **then**  6:       addSubscriber(tp_Id, brk_Id, scr_Tb);  7:       p_Brk = findIntraBrokers(tp_Id, cls_Id, pls_Tb);  8:       inform_Bridging(tp_Id, brk_Id, p_Brk);  9:    **else**10:       addSubscriber(tp_Id, brk_Id, clsH_Id, scr_Tb);11:       rl_Brk = findInterBrokers(tp_Id, scr_Tb, cls_Tb);12:       inform_Bridging(tp_Id, rlCB_Id, rl_Brk);13:       inform_Bridging(tp_Id, clsH_Id, rlCB_Id);14:       inform_Bridging(tp_Id, brk_Id, clsH_ID);15:    **end if**16:**end if**


### 4.2. Bridging Joint Subscribe Topic Channels among Broker Clusters

**Figure 4:**
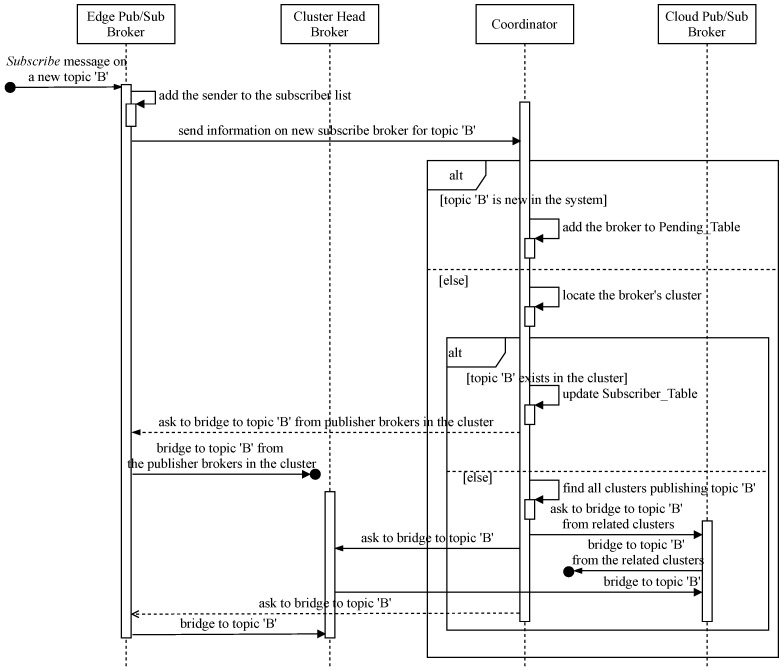
Sequence diagram for bridging joint Subscribe topic channels.

In this subsection, we describe how to link joint Subscribe topic channels among distributed brokers in our hierarchical pub/sub system. Similar to Publish, when receiving a new Subscribe message on a new topic from its clients, the responsible broker is compelled to receive all event notifications from all of the topic’s data producers that may belong to different clusters in the system to forward the germane data to interested clients. Therefore, it is necessary to coordinate all related brokers to fulfill this task under instructions from the coordinator. Figure 4 shows the sequence diagram for this coordinative procedure, and the coordinator implements Algorithm 2 to handle the Subscribe bridging process. The main interactions between involved brokers and coordination servers are described as follows:

- When a pub/sub client subscribes to new topic *B*, its broker needs to promptly inform the coordinative server to trigger joining the Subscribe topic channel process.
- The coordinator checks for any publishers of the topic in the system. If there are no publishers for the topic, the coordinator puts the requesting broker on a waiting list, called Pending_Table, and the broker will be notified later when a new publisher of the topic exists.
- If topic *B* already has publishers in the system, the coordinator needs to determine the cluster the new subscribe broker belongs to and must ascertain whether the topic’s publishers exist in that cluster or not to retrieve the cluster’s information, such as the cluster head locator, the relay cloud broker identification, etc. If the topic’s publishers already exist in the cluster, the new subscribe broker just needs to subscribe to the topic channel from all intra-cluster publish brokers with the coordinative information received from the coordinator. The coordinator adds the new member to the Subscriber_Table in the coordination database. Otherwise, the informing broker’s cluster has the first subscriber to topic *B*, but some other clusters might already have that topic’s publishers, so the coordinator asks the new subscribe cluster to join the topic *B* subscribe channel. This is obtained by retrieving from the coordination database all clusters publishing topic *B* and navigating to their corresponding relay cloud brokers. Then, the coordinator asks the new subscribe cluster to bridge to the existing channels of topic *B*. The relay cloud broker subscribes to the topic from other relay publishing brokers. The cluster head subscribes to topic *B* from the relay broker in order to forward related messages to the new broker. In turn, the new subscribe broker needs to subscribe to topic *B* from its cluster head to receive all relevant event notifications afterward.

## 5. Performance Evaluations and Discussion

In this section, we describe simulation scenarios and present experiment results to verify the correctness of the proposed model and algorithms. We implemented our hierarchical pub/sub system using Python and SimPy [42], a process-based discrete-event simulation framework. We modeled pub/sub clients and brokers as processes in SimPy, which are defined by Python generator functions. These pub/sub components interact with each other according to the coordinative protocols presented in Section 4. In our experiments, we used approximately 3350 Starbucks store locations (latitude and longitude) belonging to different cities in the United States of America as a coordinate pool for pub/sub brokers [43]. We assume the round-trip time (RTT) delay between any two brokers is a random variable with the mean is proportional to their geographical distance. Furthermore, we assume that RTTs between two cloud brokers are uniformly distributed with U[1,2] milliseconds (ms). We also assume the RTTs between IoT devices and their responsible edge brokers are uniformly distributed with an average delay of 4 ms. In all our simulation scenarios, each pub/sub broker is responsible for forwarding a list of topics, in which 80% of the topics are initialized in the bootstrap period, and the other 20% are chosen at run time. We instantiated one publisher and 10 subscribers per topic for each broker. Each publisher produced a 64-byte message in every simulation time unit (TU), and each simulation run time lasted 15 time units, in which we reserved five TUs at the beginning for topic selection and bootstrap procedures, and counted messages for the last 10 TUs. Here, we define three essential metrics to evaluate our proposal in different scenarios and to compare it with other related approaches.

- Average Delivery Latency (ADL): This value is calculated based on end-to-end message delivery delay from publishers/producers to subscribers/consumers on all tested topic channels in each scenario:
- Average Forwarding Traffic (AFT): To link joint topic channels, pub/sub brokers need to forward data packets of the channels to each other. The AFT value is defined as the ratio of total number of data packets forwarded among brokers to all data packets sent by brokers. This value helps in assessing the efficiency of bridging topics among brokers in the system.
- Node Degree (ND): A broker’s node degree is the number of connections to other brokers needed to exchange data on joint topic channels. This value reflects the resources brokers use to maintain topic overlays for data delivery.

### 5.1. Evaluation of Scaling the Topic Subscriptions

In this simulation scenario, we varied the number of topics for processing/forwarding by each pub/sub broker in order to observe the performance through the essential metrics mentioned above (ADL, AFT, and ND). We deployed 100 brokers with coordinates randomly selected from the location pool; we also created a pool comprising 1000 topics. Each broker handled 100, 150, and 200 topics for each simulation run time. These topics, selected from the 1000-topic pool in a distribution following Zipf’s law, were sent to each broker.

**Figure 5:**
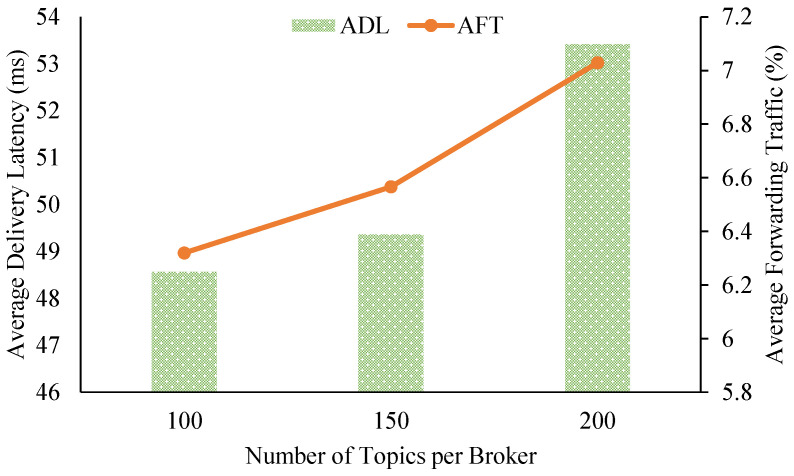
Data delivery latency and relay traffic from varying the number of topic subscriptions.

Figure 5 shows the impact on the overall delivery latency and relay traffic of the system from varying the number of topics per pub/sub broker. When we increased the topic workload per broker from 100 to 150 to 200 topics, the ADL slightly rose from about 48.56 ms to 49.36 ms to 53.42 ms, respectively. Similarly, AFT experienced a small increase from about 6.32% up to 7.03% of the forwarding traffic of all brokers. The numbers for ADL and AFT prove that brokers in our proposed pub/sub system can maintain low delivery latency and small amounts of relay traffic while scaling up the workloads of the brokers.

**Figure 6:**
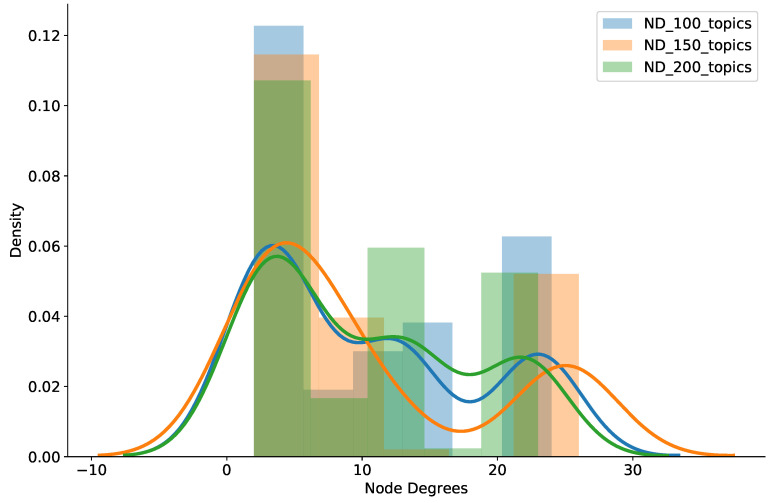
Histogram and density of node degrees from varied numbers of topics.

We also counted the brokers’ node degree (ND) in the experiment to assess how well the system worked in terms of scalable node degrees. Figure 6 shows the histogram and probability density of broker node degrees while scaling up the workloads of the pub/sub brokers. As clearly shown in the figure, our proposal maintains low node degrees quite well, even though the topic workload was expeditiously raised from 100 to 150 to 200 topics per broker. Specifically, a large percentage of brokers had a node degree of 10 or less, but most brokers had a node degree under 25, and just a small number of brokers experienced a node degree above 30. Therefore, together with low delivery latency and small amounts of relay traffic among brokers, we affirmed that our clustering strategy can provide scalable node degrees when increasing brokers’ workloads. This is very useful for deploying pub/sub systems to support large-scale IoT applications.

### 5.2. Evaluation of a Multi-Modal Topic Subscription Model

To evaluate the effect of topic correlations among brokers on the event notification performance of the pub/sub system, we implemented a multi-modal topic subscription model inspired by Wong et al. [44]. In this experiment, we instantiated 100 brokers with their locations randomly selected from the coordinate pool. We also created a 1000-topic pool to set 100 topics per broker as their topic workloads for this scenario. We varied topic correlations generated by the multi-modal model among the brokers to observe their data delivery performance as follows:

- The topic space was divided into *n* categories, or modes.
- Each broker uniformly chose *p* modes out of *n* at random.
- The ratios for p/n were fixed at 0.1 and 0.2; we varied *p* and *n* to change topic correlation levels. Specifically, with p=1, n=[10, 5] and with p=5, n=[50, 25].
- One hundred topics were selected from these *p* categories, distributed following Zipf’s law (distribution parameter a=1.2), for each broker.

**Figure 7:**
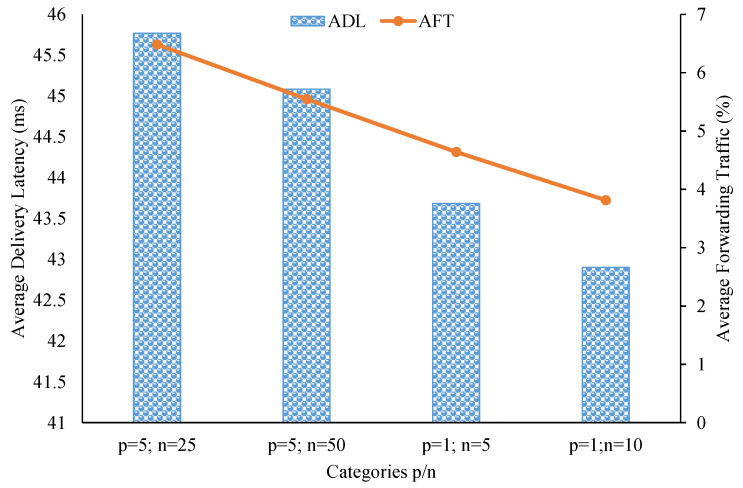
Delivery latency and relay traffic in the multi-modal model experiment.

As seen in Figure 7, both ADL and AFT decreased when topic correlations between brokers increased in the simulation. Note that in this multi-modal model setup, the most correlated case was when p=1 and n=10, and the least correlated case was when p=5 and n=25. Specifically, in the best case, ADL was 42.9 ms and AFT was about 3.81%. With these low delivery-latency and small relay-traffic values, we can justify saying our clustering scheme can take advantage of topic similarities and proximity locations among brokers to efficiently cluster them for data dissemination. However, we need to assess the brokers’ node degrees to confirm that the proposed system can function well without overloading pub/sub brokers.

**Figure 8:**
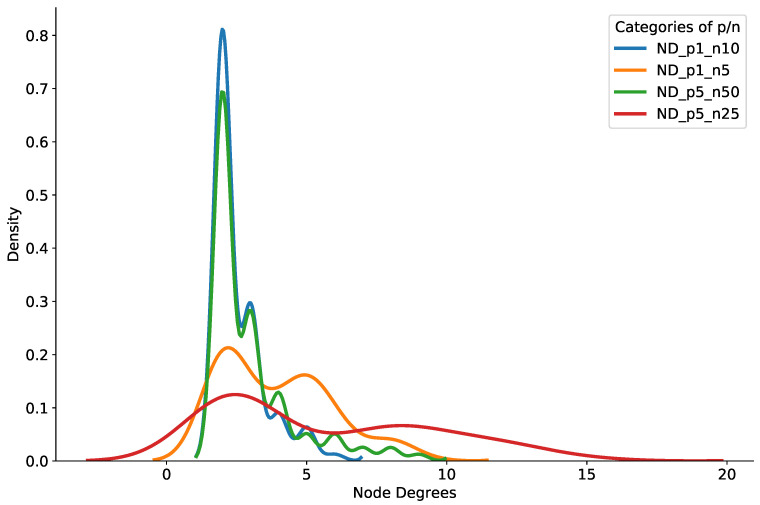
Density of brokers’ node degrees with multi-modal topic subscriptions.

Figure 8 shows the probability density of the brokers’ node degrees in the multi-modal model setup. In Figure 8, a majority of brokers had a node degree less than 10 in all of the multi-modal topic subscription modes. In addition, it is clear from the figure that only a handful of brokers had a node degree more than 15 and less than 20. We therefore assert that our pub/sub system can support low delays and provide little relay traffic, along with scalable brokers’ node degrees in the experiment settings.

### 5.3. Scalability Evaluation

This subsection presents the scalability results of our pub/sub scheme and compares them with other schemes sharing approaches akin to it. The scalability experiment consisted of multiple simulation run times, where the number of brokers increased from 100 to 500 in increments of 100. The coordinates of these brokers were randomly selected from the coordinate pool mentioned previously. Each broker was responsible for 100 topic channels, in which clients selected 70% of the topics locally and chose the other 30% from outside publishing brokers in a distribution following Zipf’s law. By doing it this way, we observed the capability of the pub/sub systems in dealing with many-to-many communications among the publishers and subscribers belonging to distributed brokers. We collected approximately 290 million end-to-end messages to measure performance metrics, i.e., delivery latency (ADL), relay traffic (AFT), and brokers’ node degrees (ND).

**Figure 9:**
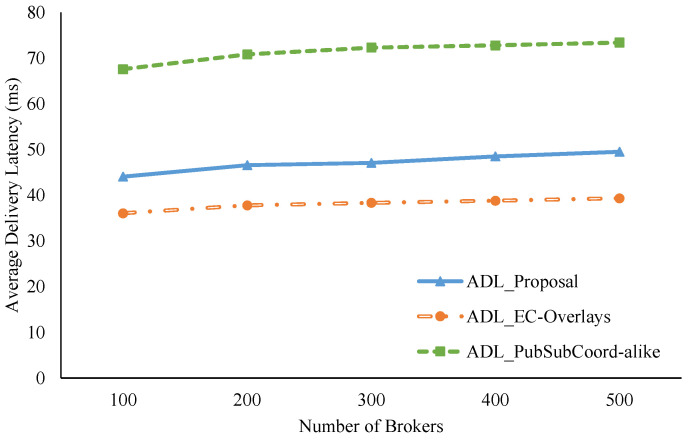
ADL comparison with increasing numbers of brokers.

**Figure 10:**
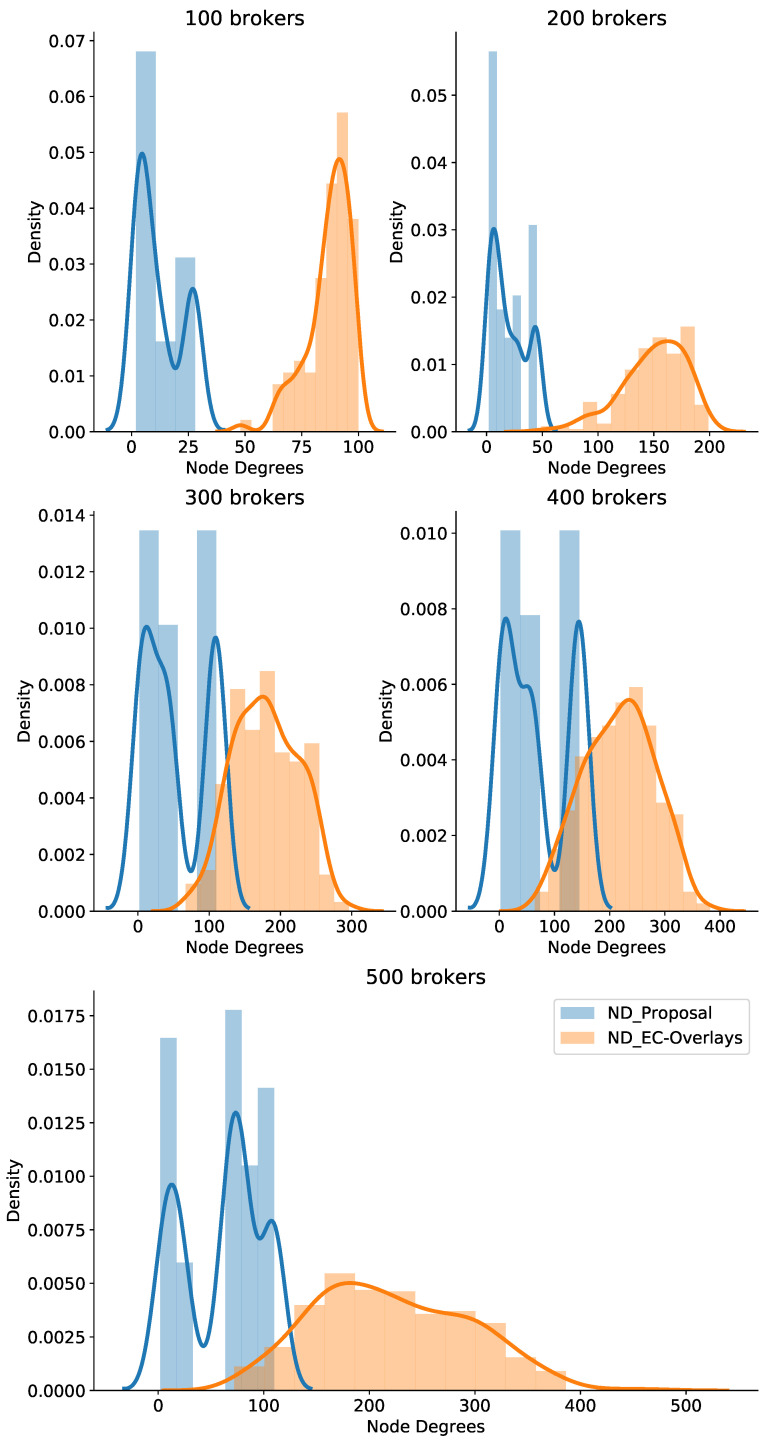
Node degrees when varying the number of brokers.

**Figure 11:**
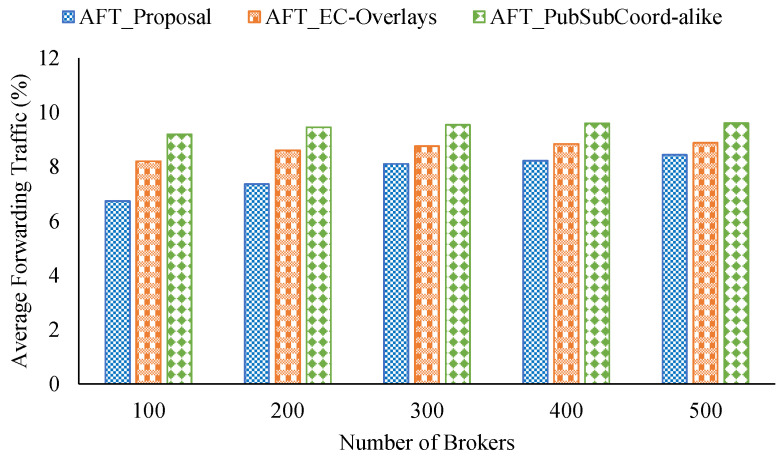
Relay traffic from varying the number of brokers.

For comparison with other similar approaches, we implemented an Edge-Cloud pub/sub scheme motivated by An et al. [29] known as PubSubCoord-alike. In this scheme, edge brokers deployed in isolated networks handle data delivery for adjacent pub/sub clients, and a group of cloud brokers manages the routing service so edge brokers can link to all topic channels of the system. We assumed that the RTTs between edge brokers and cloud brokers are uniformly distributed, where the mean delay is the average delay among edge brokers calculated from the coordinate pool. In addition, we compared the new proposal with our previous Edge-Cloud pub/sub overlay work [41] called EC-Overlays. In EC-Overlays, there is an overlay network for each topic, which includes many publish broker clusters forwarding topics’ published events, and the cluster heads connect to each other to link to the topic as a single domain for data delivery. Figure 9, Figure 10 and Figure 11 show the experiment results.

As shown in Figure 9, our proposal has ADLs much better than the PubSubCoord-alike scheme in all test cases. Specifically, our proposal’s ADL, on average, is approximately 66% of PubSubCoord-alike’s ADL. In the test case with 500 brokers, for example, our ADL value is 49.49 ms while that of PubSubCoord-alike is 73.38 ms. Our proposal achieves superior ADL results because the clustering procedures can take advantage of topic similarities and geolocation proximity among edge brokers to congregate them into fitting clusters. As a result, edge brokers can directly exchange joint topic data inside a cluster to reduce latency. Conversely, in the PubSubCoord-alike approach, data exchanged among edge brokers is usually sent and received through a cloud broker. Therefore, it causes sizeable delays for end-to-end data delivery. Furthermore, as clearly shown in Figure 9, although we increased the number of brokers from 100 to 500 by 100 each time, our proposal’s ADL experienced just a trivial rise, from 44.05 ms to 49.49 ms.

Although our method attained great results in terms of ADL, the EC-Overlays method achieved even better results. In addition, EC-Overlays prevailed over the other approaches in all test cases. In particular, the EC-Overlays’ ADL was merely 36.05 ms and 39.31 ms for test cases with 100 and 500 brokers, respectively. The reason for this excellent result is that the EC-Overlays approach takes advantage of many direct connections between edge brokers to serve up data transmissions. Consequently, the pub/sub brokers in EC-Overlays may consume much of the resources in order to maintain topic overlay networks, which can cause scalability problems for the system. Therefore, we counted ND in the experiment to evaluate this issue. Figure 10 plots the broker node degrees in our proposal and EC-Overlays when varying the number of brokers. From the graph, it is clear that EC-Overlays had very high broker node degrees in all test cases. That means brokers in EC-Overlays need to consume a lot of resources to maintain the topic overlay networks for data delivery. Take the case of 500 brokers, for example—most of the brokers in EC-Overlays experienced node degrees of more than 150, whereas our proposal’s broker NDs were less than 100. In short, our proposal strikes a good balance between data delivery latency and system scalability. This is an extremely important feature for IoT systems functioning on a large scale.

The relay traffic performance of the three approaches when varying the number of brokers is shown in Figure 11. We observe that our proposal performed the best in terms of AFT in all cases considered, about 7.77% on average. By contrast, PubSubCoord-alike scored the worst in all the cases examined. For example, with 300 brokers, our proposal’s AFT was 8.08% while PubSubCoord-alike’s was 9.54%.

## 6. Conclusions

This paper presented a hierarchical Edge-Cloud pub/sub brokers model to support data delivery with low latency and high scalability for wide-scale IoT systems. In our model, we apply implicit collaborative filtering techniques to predict future topics of brokers in order to proactively cluster brokers based on topic similarities and proximity. Then, topic channel routing is conducted via a two-layer routing scheme, i.e., intra-cluster and inter-cluster procedures. Inside a cluster, pub/sub brokers directly exchange data with each other. For joint topics among broker clusters, cluster heads route event notifications to their relay cloud brokers for data delivery among the clusters involved.

Simulation results prove that our proposed model can provide data delivery with low latency and small amounts of relay traffic while maintaining scalable broker node degrees under a variety of simulation settings, such as varying the number of topic subscriptions and dealing with multi-modal topic subscriptions. In comparison with other similar approaches, our proposal outperformed PubSubCoord-alike in terms of both latency and relay traffic. In addition, our solution achieved balance among ADL, AFT, and ND, which is critical for system scalability when applied to wide-scale IoT applications.

Although our simulation-based experiments verify and validate the correctness of the proposed model and algorithms, additional cost metrics need to be considered, such as computing, storage, and networking costs. Along with these metrics’ consideration, we will develop the hierarchical brokers model on an emulated Edge-Cloud testbed and provide an efficient solution of the best-fit cost-performance trade-off for wide-scale IoT application deployments in our future work.

## Figures and Tables

**Table 1:** Variables used in Algorithms 1 and 2 and their brief descriptions.

Variable Name	Description
tp_Id	Topic identification
brk_Id	Broker identification
pls_Tb	Publisher_Table, stores information on topics’ publish brokers
scr_Tb	Subscriber_Table, stores information on topics’ subscribe brokers
pd_Tb	Pending_Table, stores information on topics’ pending brokers
cls_Tb	Cluster_Table, stores a cluster’s information: cluster head, cluster members,
	publish/subscribe topics, cluster relay broker
cls_Id	Cluster identification
rlCB_Id	Relay cloud broker identification
clsH_Id	Cluster head identification
pd_Scr	Saves information on pending subscribers to a topic
p_Brk	Peer brokers, saves intra-brokers of a topic
rl_Brk	Relay brokers, saves topics’ relay brokers

## Abbreviations

The following abbreviations are used in this manuscript:

IoT	Internet of Things
PSP	Publish/Subscribe Paradigm
TBPS	Topic-based Publish/Subscribe
MQTT	Message Queue Telemetry Transport
API	Application Programming Interface
MEC	Mobile Edge Computing
RL	Reinforcement Learning
FCM	Firebase Cloud Messaging
CF	Collaborative Filtering
RS	Recommender System
SVD	Singular Value Decomposition
ALS	Alternate Least Squares
RTT	Round-Trip Time
TU	Time Unit
ADL	Average Delivery Latency
AFT	Average Forwarding Traffic
ND	Node Degree

## References

[1] Gubbi J., Buyya R., Marusic S., Palaniswami M. (2013). Internet of things (iot): A vision, architectural elements and future directions. Future Gener. Comput. Syst..

[2] Al-Fuqaha A., Guizani M., Mohammadi M., Aledhari M., Ayyash M. (2015). Internet of Things: A Survey on Enabling Technologies, Protocols, and Applications. IEEE Commun. Surv. Tutor..

[3] Mell P., Grance T. (2011). The NIST Definition of Cloud Computing.

[4] Bonomi F., Milito R., Zhu J., Addepalli S. (2012). Fog computing and its role in the internet of things. Proceedings of the First Edition of the MCCWorkshop on Mobile Cloud Computing.

[5] Kolozali Ş., Bermudez-Edo M., FarajiDavar N., Barnaghi P., Gao F., Ali M.I., Mileo A., Fischer M., Iggena T., Kuemper D. (2018). Observing the pulse of a city: A smart city framework for real-time discovery, federation, and aggregation of data streams. IEEE Internet Things J..

[6] Pavlopoulou N., Curry E. (2021). IoTSAX: A Dynamic Abstractive Entity Summarisation Approach with Approximation and Embedding-based Reasoning Rules in Publish/Subscribe Systems. IEEE Internet Things J..

[7] Dhelim S., Ning H., Aung N. (2021). ComPath: User interest mining in heterogeneous signed social networks for Internet of people. IEEE Internet Things J..

[8] Ramachandran G.S., Krishnamachari B. (2019). Towards a large scale iot through partnership, incentive, and services: A vision, architecture, and future directions. Open J. Internet Things (OJIOT).

[9] Almajali S., Dhiah el Diehn I., Salameh H.B., Ayyash M., Elgala H. (2019). A distributed multi-layer MEC-cloud architecture for processing large scale IoT-based multimedia applications. Multimed. Tools Appl..

[10] Eugster P.T., Felber P.A., Guerraoui R., Kermarrec A.M. (2003). The many faces of publish/subscribe. ACM Comput. Surv. CSUR.

[11] Rahimian F., Girdzijauskas S., Payberah A.H., Haridi S. Vitis: A Gossip-based Hybrid Overlay for Internet-scale Publish/Subscribe Enabling Rendezvous Routing in Unstructured Overlay Networks. Proceedings of the 2011 IEEE International Parallel & Distributed Processing Symposium.

[12] Setty V., Kreitz G., Vitenberg R., Van Steen M., Urdaneta G., Gimåker S. (2013). The hidden pub/sub of spotify: (Industry article). Proceedings of the 7th ACM International Conference on Distributed Event-Based Systems.

[13] Antonic A., Roankovic K., Marjanovic M., Pripuic K., Arko I.P. A Mobile Crowdsensing Ecosystem Enabled by a Cloud-Based Publish/Subscribe Middleware. Proceedings of the 2014 International Conference on Future Internet of Things and Cloud.

[14] Rowe A., Berges M.E., Bhatia G., Goldman E., Rajkumar R., Garrett J.H., Moura J.M., Soibelman L. (2011). Sensor Andrew: Large-scale campus-wide sensing and actuation. IBM J. Res. Dev..

[15] Menzel T., Karowski N., Happ D., Handziski V., Wolisz A. Social sensor cloud: An architecture meeting cloud-centric iot platform requirements. Proceedings of the 9th KuVS NGSDP Expert Talk on Next Generation Service Delivery Platforms.

[16] Message Queue Telemetry Transport. http://mqtt.org/.

[17] Chen C., Tock Y., Jacobsen H., Vitenberg R. Weighted Overlay Design for Topic-Based Publish/Subscribe Systems on Geo-Distributed Data Centers. Proceedings of the 2015 IEEE 35th International Conference on Distributed Computing Systems.

[18] Shi Y., Zhang Y., Jacobsen H.-A., Tang L., Elliott G., Zhang G., Chen X., Chen J. (2019). Using Machine Learning to Provide Reliable Differentiated Services for IoT in SDN-Like Publish/Subscribe Middleware. Sensors.

[19] Mohammadi M., Al-Fuqaha A. (2018). Enabling cognitive smart cities using big data and machine learning: Approaches and challenges. IEEE Commun. Mag..

[20] Rathore M.M., Ahmad A., Paul A., Rho S. (2016). Urban planning and building smart cities based on the internet of things using big data analytics. Comput. Netw..

[21] Martins J. (2018). Towards Smart City Innovation under the Perspective of Software-Defined Networking, Artificial Intelligence and Big Data. arXiv.

[22] Zhao L., Wang J., Liu J., Kato N. (2019). Routing for Crowd Management in Smart Cities: A Deep Reinforcement Learning Perspective. IEEE Commun. Mag..

[23] Arruda C.E., Moraes P.F., Agoulmine N., Martins J.S. (2020). Enhanced Pub/Sub Communications for Massive IoT Traffic with SARSA Reinforcement Learning. Proceedings of the International Conference on Machine Learning for Networking.

[24] Firebase Cloud Messaging. https://firebase.google.com/docs/cloud-messaging/.

[25] IoTivity Software Framework. https://iotivity.org/.

[26] Girdzijauskas S., Chockler G., Vigfusson Y., Tock Y., Melamed R. Magnet: Practical subscription clustering for internet-scale publish/subscribe. Proceedings of the 4th ACM International Conference on Distributed Event-Based Systems (DEBS).

[27] Chockler G., Melamed R., Tock Y., Vitenberg R. (2007). Spidercast: A scalable interest-aware overlay for topic-based pub/sub communication. Proceedings of the 2007 Inaugural International Conference on Distributed Event-Based Systems.

[28] Gascon-Samson J., Garcia F., Kemme B., Kienzle J. Dynamoth: A Scalable Pub/Sub Middleware for Latency-Constrained Applications in the Cloud. Proceedings of the 2015 IEEE 35th International Conference on Distributed Computing Systems.

[29] An K., Khare S., Gokhale A., Hakiri A. An autonomous and dynamic coordination and discovery service for wide-area peer-to-peer publish/subscribe: Experience paper. Proceedings of the 11th ACM International Conference on Distributed and Event-Based Systems.

[30] Happ D. (2018). Cloud and fog computing in the internet of things. Internet of Things A to Z: Technologies and Applications.

[31] Yu W., Liang F., He X., Hatcher W.G., Lu C., Lin J., Yang X. (2017). A survey on the edge computing for the Internet of Things. IEEE Access.

[32] Koren Y., Bell R. (2015). Chapter Advances in Collaborative Filtering. Recommender Systems Handbook.

[33] Hu Y., Koren Y., Volinsky C. Collaborative filtering for implicit feedback datasets. Proceedings of the 2008 Eighth IEEE International Conference on Data Mining.

[34] Rossetti M., Stella F., Zanker M. Towards explaining latent factors with topic models in collaborative recommender systems. Proceedings of the 2013 24th International Workshop on Database and Expert Systems Applications.

[35] Koren Y. (2010). Factor in the neighbors: Scalable and accurate collaborative filtering. ACM Trans. Knowl. Discov. Data.

[36] Pan R., Scholz M. Mind the gaps: Weighting the unknown in large-scale one-class collaborative filtering. Proceedings of the 15th ACM SIGKDD International Conference on Knowledge Discovery and Data Mining.

[37] Paterek A. Improving regularized singular value decomposition for collaborative filtering. Proceedings of the KDD Cup and Workshop.

[38] Takács G., Pilászy I., Németh B., Tikk D. (2007). Major components of the gravity recommendation system. ACM Sigkdd Explor. Newsl..

[39] Campello R.J., Moulavi D., Sander J. (2013). Density-based clustering based on hierarchical density estimates. Proceedings of the Pacific-Asia Conference on Knowledge Discovery and Data Mining.

[40] Zhao Y., Kim K., Venkatasubramanian N. (2013). Dynatops: A dynamic topic-based publish/subscribe architecture. Proceedings of the 7th ACM International Conference on Distributed Event-Based Systems.

[41] Pham V.-N., Nguyen V., Nguyen T.D.T., Huh E.-N. (2020). Efficient Edge-Cloud Publish/Subscribe Broker Overlay Networks to Support Latency-Sensitive Wide-Scale IoT Applications. Symmetry.

[42] SimPy. https://simpy.readthedocs.io/en/latest/.

[43] Starbucks Store Location Data. https://data.world/data-hut/starbucks-store-location-data.

[44] Wong T., Katz R., Mccanne S. An evaluation of preference clustering in large-scale multicast applications. Proceedings of the IEEE INFOCOM.

